# Robotic-based carbon ion therapy and patient positioning in 6 degrees of freedom: setup accuracy of two standard immobilization devices used in carbon ion therapy and IMRT

**DOI:** 10.1186/1748-717X-7-51

**Published:** 2012-03-29

**Authors:** Alexandra D Jensen, Marcus Winter, Sabine P Kuhn, Jürgen Debus, Olaf Nairz, Marc W Münter

**Affiliations:** 1 Dept of Radiation Oncology, University of Heidelberg, INF 400, Heidelberg 69120, Germany; 2 Dept of Medical Physics, Heidelberg Ion Beam Therapy Centre (HIT), Heidelberg, Germany

## Abstract

**Purpose:**

To investigate repositioning accuracy in particle radiotherapy in 6 degrees of freedom (DOF) and intensity-modulated radiotherapy (IMRT, 3 DOF) for two immobilization devices (Scotchcast masks vs thermoplastic head masks) currently in use at our institution for fractionated radiation therapy in head and neck cancer patients.

**Methods and materials:**

Position verifications in patients treated with carbon ion therapy and IMRT for head and neck malignancies were evaluated. Most patients received combined treatment regimen (IMRT plus carbon ion boost), immobilization was achieved with either Scotchcast or thermoplastic head masks. Position corrections in robotic-based carbon ion therapy allowing 6 DOF were compared to IMRT allowing corrections in 3 DOF for two standard immobilization devices. In total, 838 set-up controls of 38 patients were analyzed.

**Results:**

Robotic-based position correction including correction of rotations was well tolerated and without discomfort. Standard deviations of translational components were between 0.5 and 0.8 mm for Scotchcast and 0.7 and 1.3 mm for thermoplastic masks in 6 DOF and 1.2 - 1.4 mm and 1.0 - 1.1 mm in 3 DOF respectively. Mean overall displacement vectors were between 2.1 mm (Scotchcast) and 2.9 mm (thermoplastic masks) in 6 DOF and 3.9 - 3.0 mm in 3 DOF respectively. Displacement vectors were lower when correction in 6 DOF was allowed as opposed to 3 DOF only, which was maintained at the traditional action level of > 3 mm for position correction in the pre-on-board imaging era.

**Conclusion:**

Setup accuracy for both systems was within the expected range. Smaller shifts were required when 6 DOF were available for correction as opposed to 3 DOF. Where highest possible positioning accuracy is required, frequent image guidance is mandatory to achieve best possible plan delivery and maintenance of sharp gradients and optimal normal tissue sparing inherent in carbon ion therapy.

## Introduction

High-precision radiotherapy has raised the interest in positioning systems allowing patient positioning in more than three degrees of freedom (3DOF). Initial investigations have been carried out using the automated HexaPOD in combination with MV-cone-beam CT online correction [1,2], and some particle therapy centers have reported experiences with robotic-based treatment tables also enabling positioning in six degrees of freedom (6 DOF) [3,4]. In high-precision techniques and even more so in particle therapy, higher degrees of freedom offer various advantages over standard treatment tables. First, 6 DOF allow higher flexibility in treatment planning and choice of beam angles particularly in treatments with fixed beam lines. Second, patient positioning is a crucial issue in particle therapy due to the highly conformal dose distributions obtained by scanned particle beams. Integrity of planned dose distributions largely depends on set-up accuracy and reproducibility of patient position; hence set-up variations may cause considerable range uncertainties. Image guidance and subsequent position correction in 6 DOF promise further optimization of patient positioning as opposed to 3 DOF. Third, it may also be a valuable tool once tracking of moving targets finds clinical application in particle therapy.

Traditionally, various immobilization devices for fractionated radiotherapy have been tested with regard to their repositioning accuracy. Mouthpiece- or bite-plate-based masks yield precisions of 0.5 - 1 mm [5,6]. Albeit highly precise, these masks are less feasible for patients with head and neck malignancies faced with the often times poor dental status and increasing discomfort caused by radiation-induced mucositis. Hence, the most widely used, non-invasive immobilization devices include thermoplastic material either with or without shoulder fixation yielding a repositioning accuracy of between 0.9 mm and 3.4 mm [7-16]. However, Scotchcast custom-made solutions are sometimes used and showed comparatively small set-up errors of 1.8 mm for intracranial targets [17] and 3.1 - 5.7 mm for extracranial targets within the head and neck depending on isocentre localization [18,19]. The remaining set-up uncertainties demand an increasing use of image guidance. Results of Zeidan et al [20] could demonstrate residual setup errors in fractionated RT to decrease as frequencies of image guidance increases. As a consequence especially for techniques mandating the highest possible level of positioning accuracy such as particle therapy, frequent image guidance is compulsory.

The Heidelberg Ion Therapy center (HIT) is equipped with both a robotic table and robotic C-arm in both horizontal treatment rooms. The purpose of this study was to investigate interfractional positioning accuracy when position correction in 6 DOF is allowed (using the robotic table in particle therapy) compared to standard position correction in 3 DOF with a standard treatment table as used in intensity-modulated radiotherapy (IMRT). In addition, two immobilization devices currently used for fractionated radiation therapy of head and neck cancer patients at our institution were evaluated in this setting.

## Materials and methods

Position controls of radiotherapy treatment in patients undergoing either combined IMRT plus carbon ion boost or C12 only for head and neck cancer were collected and analyzed. Most patients were treated for malignant salivary gland tumors (MSGT); this series however, also includes malignant melanoma and paranasal sinus malignancies. The majority of patients underwent combined treatment protocols (IMRT plus carbon ion boost) as a primary treatment, a few patients received carbon ion therapy only for re-irradiation. For this analysis, 38 patients (median age: 56 years [range 23 - 78 years]) with a total of 838 individual setup controls (308 in 6 DOF, 530 in 3 DOF) were evaluated for the treatment period from 11/2009 to 07/2010.

Extension of target volumes and therefore isocentre positions were similar in all patients. Patient baseline characteristics are included in Table 1.

**Table 1:** Baseline characteristics

radiation therapy	dose (Gy/GyRBE)	range (Gy/GyRBE)	
carbon ion therapy	23.5	18 - 24.4	
IMRT	49	39 - 51	

**diagnoses**		**patients**	

adenoid cystic carcinoma		31	
malignant melanoma		3	
mucoepidermoid carcinoma		1	
nasopharyngeal carcinoma		1	
osteosarcoma		1	
adenocarcinoma		1	

	**Scotchcast mask**	**thermoplastic head mask**	**total**

**patients**	20	18	38
**number of position controls**			
6 DOF	162	146	308
3 DOF	319	211	530

### Immobilization/RT planning

**Figure 1:**
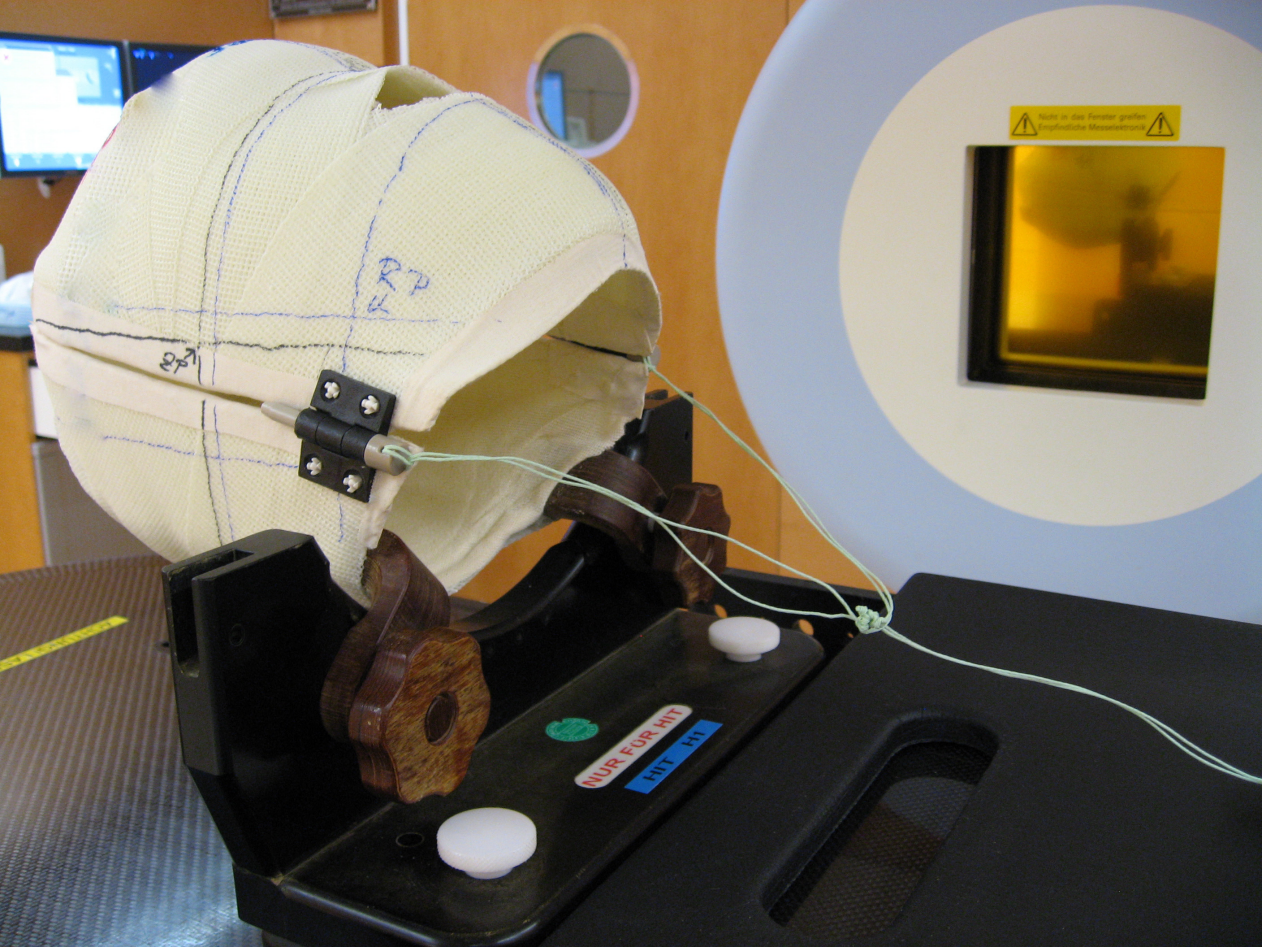
**Scotchcast mask**.

**Figure 2:**
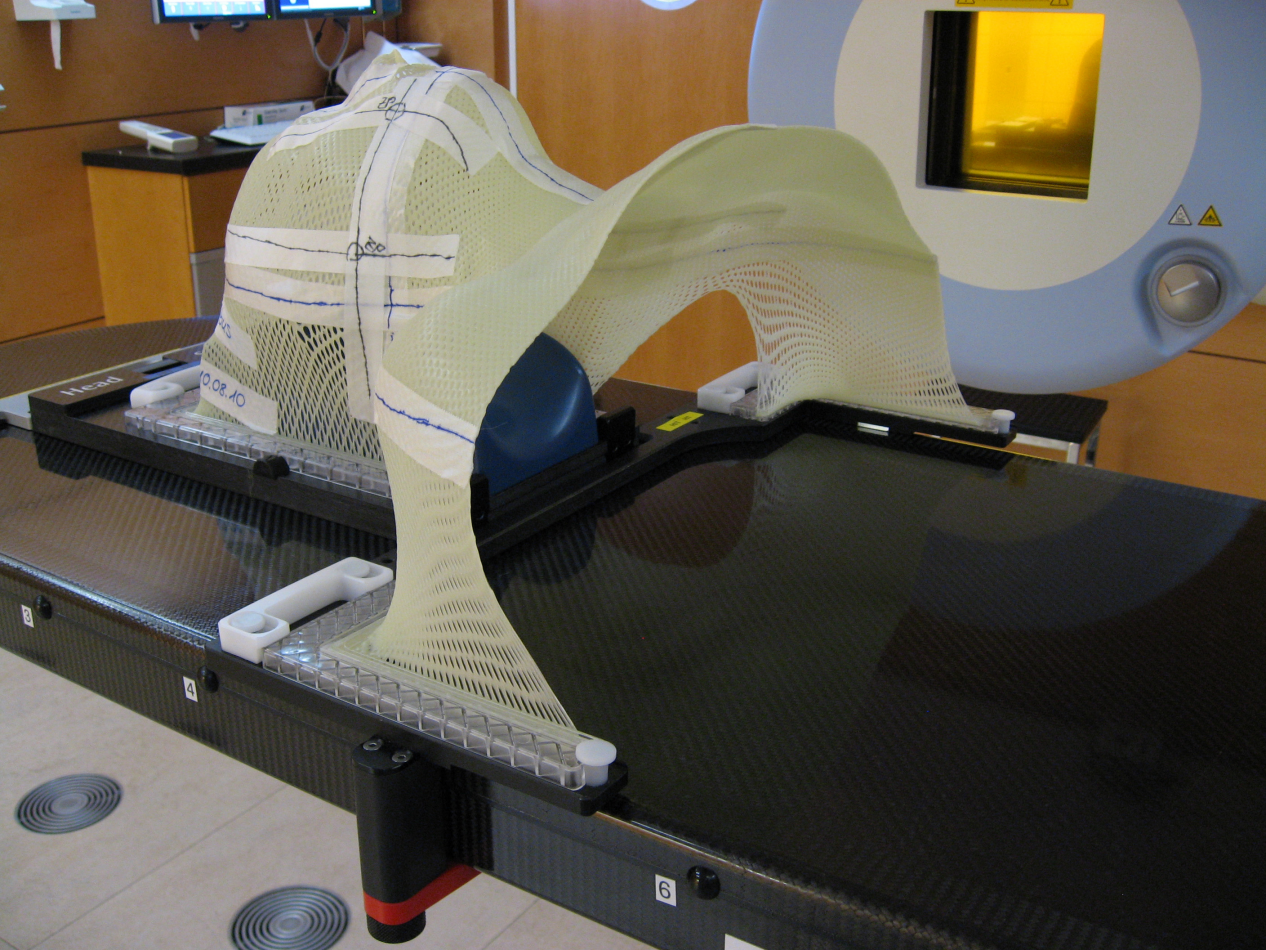
**Thermoplastic head mask including shoulder fixation**.

Two systems currently in use for precision RT in head and neck tumors were analyzed: The Scotchcast head mask (Figure 1) and the thermoplastic head mask including shoulder fixation and head rest (HeadSTEP^®^, IT-V) (Figure 2). Both systems are custom-made for each individual patient.

The Scotchcast head mask uses self-hardening bandages (Scotchcast, Scotch Flex, 3 M Co), which fix the patient to the stereotactic base frame. This system was developed in-house but can be commercially obtained through Leibinger^®^.

The thermoplastic head mask including shoulder fixation consists of a new thermoplastic material, which can be individually modeled to the patient's shape but uses a standard head-rest and table fixation for all patients (HeadSTEP^®^, IT-V). Twenty patients in this cohort were immobilized with Scotchcast masks, 18 patients with thermoplastic head masks and shoulder fixation.

Imaging for radiation treatment planning includes CT in above-mentioned set-up at 3 mm slices as well as contrast-enhanced MRI for 3D correlation and target delineation.

### Radiotherapy and target volumes/dose prescription

Carbon ion therapy was delivered with a horizontal beam line in active beam application/raster scanning technique [21] at the Heidelberg Ion Therapy Centre (HIT), IMRT was delivered at the Dept. of Radiation Oncology Heidelberg. Isocentre localization was performed in a virtual setup: the respective reference points were marked on the immobilization devices and identified on the planning scan by 3 Beekly spots. For the Scotchcast masks, the treatment isocentre was localized stereotactically. The displacement vector was calculated based on CT-coordinates for thermoplastic head masks and based on stereotactic registration in Scotchcast masks.

CTV1 (carbon ion boost) included the macroscopic tumor/prior tumor bed. Twenty-four GyRBE carbon ions are prescribed to the CTV1 in 3 GyRBE/fraction (5 fractions per week) (coverage: 95% prescription isodose). CTV2 includes CTV1 with safety margins along typical pathways of spread. In malignant salivary gland tumors, ipsilateral nodal levels (II and III) were included, additional nodal levels were covered as indicated.

Treatment isocentres for carbon ion therapy were chosen at geometrical centre of the respective CTV1 (mostly in the paranasal sinuses), treatment isocentre for IMRT (CTV2) were chosen close to the isocentre of CTV1.

Fifty Gy IMRT (inversely planned step-and-shoot technique) in 25 fractions (5 fractions per week) were prescribed to the CTV2 (coverage at least with the 90% prescription isodose) taking into account doses applied by image guidance with MV-cone-beam CT.

In the combined treatments (IMRT + C12-boost), patients received carbon ion treatment as an upfront boost before undergoing IMRT.

### Positioning/image guidance

#### Carbon ion therapy: 6 degrees of freedom (6 DOF)

The robotic-based treatment table allows patient positioning in 6 DOF. Mean radial positioning accuracy was measured to be below 0.2 mm ± 0.2 mm standard deviation for the target positions of the investigated patients. Correction of rotational errors with the robotic table is limited to a maximum of 5 degrees in patient-mode. The robot-mounted C-arm allows position verification in almost all treatment positions with a mean radial positioning accuracy of 0.2 mm ± 0.1 mm standard deviation.

**Figure 3:**
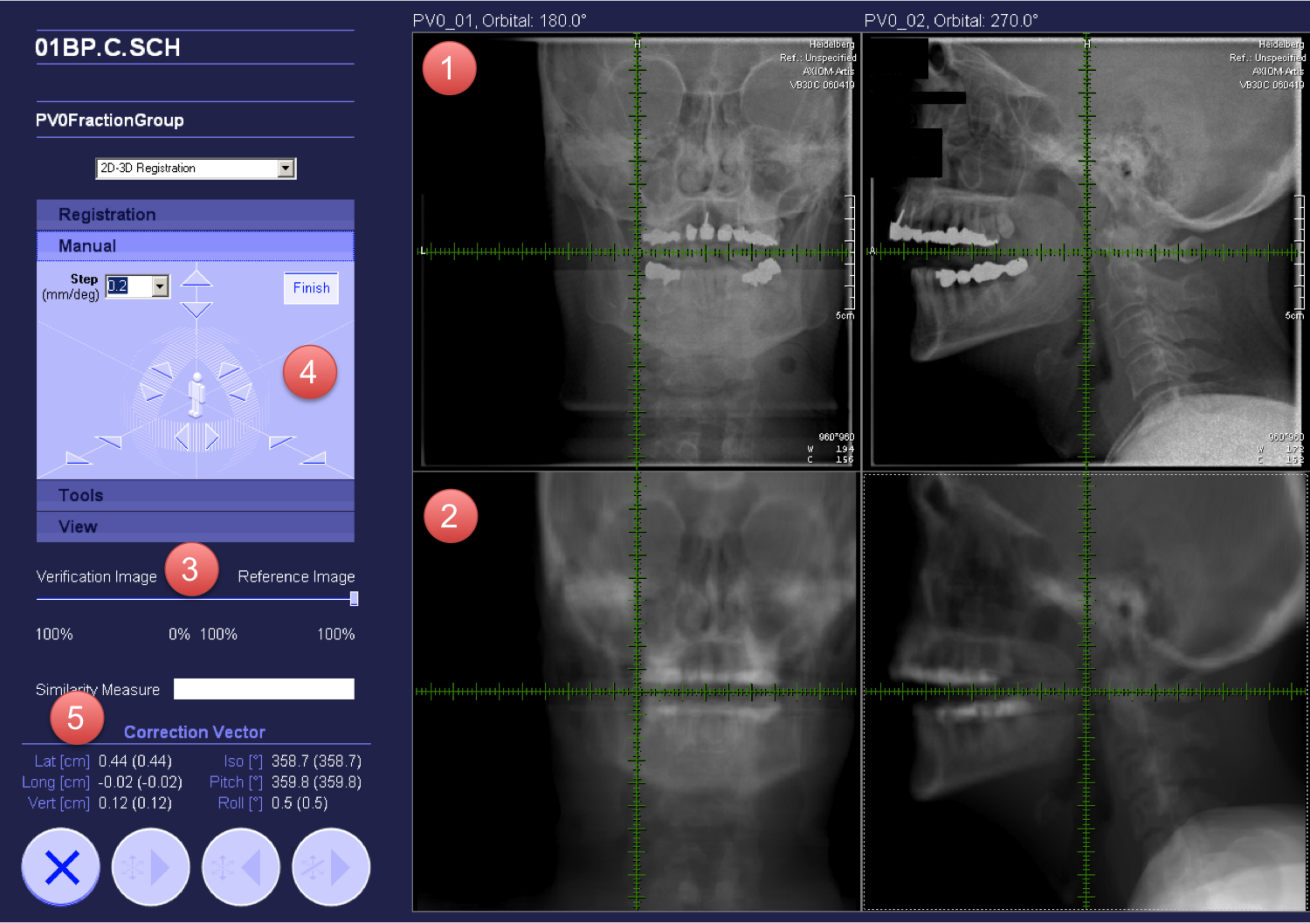
**Particle therapy position correction tool: 1: orthogonal x-rays acquired for position control at each session 2: digitally reconstructed radiographs (DRRs) from the planning CT-scan 3: slider to switch between the x-ray (position control) and DRR (planning scan)**. 4: manual correction tool for 6 DOF 5: resulting correction vector.

After acquisition of orthogonal x-rays, an automatic 2D-3D pre-match to orthogonal digitally reconstructed radiographs (DRRs) was carried out (Siemens syngo PT treatment). In this process, the daily position control is used as the reference image, DRRs are daily reconstructed by the Siemens syngo PT treatment software. By matching the DRRs to the x-ray position control, not only translational but also rotational shifts can be determined. The pre-match offered by the software is verified by the radiotherapist/radiation oncologist with regard to bony anatomy. Manual adjustment of the match was carried out on-line using the manual correction tool (Figure 3 (4)); the resulting correction vector, including rotations, was subsequently applied to the patient position (Figure 3). Patient position controls were carried out in each session and shifts were always corrected. None of the patients needed manual repositioning.

#### IMRT: 3 degrees of freedom (3 DOF)

**Figure 4:**
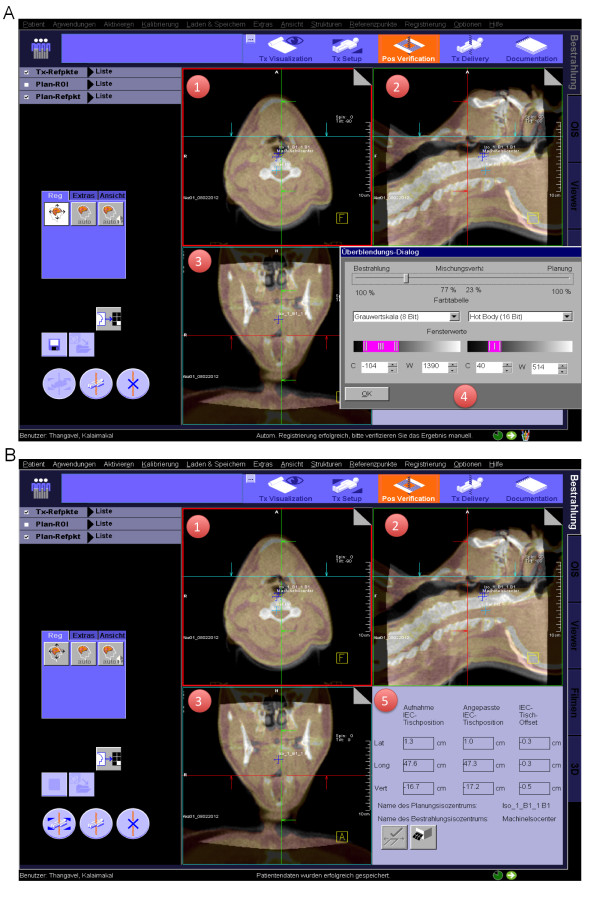
**a/b: Routine photon therapy position correction tool: 1: axial cone-beam CT/planning CT scan overlay; cone-beam CT: gray scale, planning CT: hot body 2: sagittal cone-beam CT/planning CT scan overlay 3: coronal cone-beam CT/planning CT scan overlay 4: slider to switch between the x-ray (position control) and DRR (planning scan)**. 5: resulting correction vector.

IMRT treatments were carried out at a Siemens ARTISTE using the standard treatment table with 3 DOF and Siemens syngo RT software. MV-cone-beam CTs (MV-CBCT) were used for position verification at least twice weekly. After acquisition of the MV-CBCTs, an automatic 3D-3D pre-match was carried out. Further procedure was similar as for carbon therapy: the automatic pre-match is checked and verified by the radiotherapist/radiation oncologist with regard to bony anatomy (Figure 4A). Manual adjustment of the match is carried out on-line by moving the planning scan to the desired place on the acquired cone-beam CT (drag& drop). All correction vectors in this series were applied to the patient position (Figure 4b). Current in-house procedures define an action level of 3 mm in fractionated high-precision RT- of head and neck patients. The robotic table in particle therapy moves automatically to the adjusted position, the standard table in routine photon therapy needs to be manually adjusted according to the resulting table offset given by the software.

### Data acquisition/analysis

Applied correction vectors of each position verification were analyzed for each patient. Interfractional positioning accuracy was defined as isocentre displacement (position control) according to Bentel et al. [22].

Mean/median values were calculated for 3 and 6 DOF for every patient. Lateral is defined as right to left, longitudinal as cranial to caudal, and vertical as ventral to dorsal, whereas "iso" defines the rotation around the vertical axis, "pitch" rotation around the lateral axis, and "roll" around the longitudinal axis (Figure 3).

The displacement vector was calculated for each treatment by ($\vec{v}=\sqrt{x^{2}+y^{2}+z^{2}}$, with x, y and z substituted for lateral, longitudinal and vertical shifts, respectively); also the mean and standard deviation of displacement vectors were calculated for each patient. As only isocentre shifts are considered, rotations do not contribute to the displacement vector regarding the isocentre position. Extension of target volumes and therefore isocentre positions were similar in all patients.

Positioning accuracy as defined above was evaluated for the two immobilization devices and 6 DOF vs. 3 DOF positioning correction by comparison of median translations/rotations as well as overall displacement vectors of isocentre shifts.

In order to compare random errors between the two immobilization systems, the following analysis was performed for all degrees of freedom: random errors for each patient were obtained by subtraction of the mean displacement for all setup controls of the respective patient. The random error is a measure of reproducibility of the immobilization device used [23]. Subsequently, the standard deviation σ_c _of this centered data set (including all set-up controls) was calculated. This is equivalent to calculating the root mean square of all patient random errors.

Calculations and statistical analyses were performed using the calculation tool and parametric tests of Addinsoft xlstat 2011.

## Results

838 position controls (308 in 6 DOF, 530 in 3 DOF) in 38 patients were evaluated. Most patients were treated for malignant salivary gland neoplasms, treatment isocentres were all located in the head (mostly the paranasal sinuses) though target volumes for subsequent IMRT-treatments did extend to the nodal neck levels II-III. Position verification including position correction and manual adjustment added approximately 10-15 min to the total treatment time in carbon ion therapy. Corrective table rotations in pitch and roll went up to 4.4° and were generally not perceived as uncomfortable.

Absolute overall translational and rotational corrections for each degree of freedom ranged from -3.1 mm to 4.8 mm and -2.6° to 2.4° for Scotchcast masks and from -6.1 mm to 5.3 mm and -3.2° and 4.4° for thermoplastic masks in position corrections allowing 6 DOF. Translational shifts in 3 DOF ranged between -9 mm and 9 mm for Scotchcast masks and between - 7 mm and 7 mm for thermoplastic masks.

Mean corrections and centered standard deviations for all setups are listed in Table 2.

**Table 2:** Corrections in 3 and 6 DOF; σ_c_: centered standard deviation

corrections in 6 DOF						
	**Scotchcast head mask**			**thermoplastic head mask**		
	
	**mean**	**sigma**	**p-value**	**mean**	**sigma**	**p-value**

**longitudinal (mm)**	1.2	1.3	0.046	0.0	1.9	0.836
**lateral (mm)**	-0.3	1.0	0.153	0.5	1.6	0.127
**vertical (mm)**	0.4	0.8	0.113	1.2	1.2	0.047
**iso (°)**	0.3	0.6	0.142	-0.1	0.9	0.396
**pitch (°)**	0.3	0.8	0.148	0.4	1.1	0.119
**roll (°)**	0.2	0.6	0.175	-0.2	1.2	0.258
**overall displacement vector (mm)**	2.1			2.55		
		**sigma**_c_			**sigma**_c_	

**longitudinal (mm)**		0.8			1.3	< 0.001
**lateral (mm)**		0.6			0.9	< 0.001
**vertical (mm)**		0.6			0.7	0.062
**iso (°)**		0.4			0.5	0.019
**pitch (°)**		0.5			0.7	< 0.001
**roll (°)**		0.4			0.6	< 0.001

**corrections in 3 DOF**						

	**Scotchcast head mask**			**thermoplastic head mask**		
	
	**mean**	**sigma**	**p-value**	**mean**	**sigma**	**p-value**

**longitudinal (mm)**	0.4	2.3	0.133	0.6	1.9	0.107
**lateral (mm)**	-0.4	1.9	0.122	1.1	1.8	0.055
**vertical (mm)**	-0.4	2.3	0.145	0.00	1.8	1.000
		**sigma**_c_			**sigma**_c_	

**longitudinal (mm)**		1.4			1.0	< 0.001
**lateral (mm)**		1.3			1.0	< 0.001
**vertical (mm)**		1.2			1.1	0.096
**overall displacement vector (mm)**	3.5			3.0		

In 6 DOF position corrections, centered standard deviations were slightly higher in patients with thermoplastic masks reaching statistical significance in the lateral and longitudinal component. In 3 DOF centered standard deviations showed statistically significant differences for the lateral and longitudinal components.

The corresponding mean overall displacement vectors were calculated to 2.1 mm (Scotchcast) and 2.55 mm (thermoplastic) in 6 DOF and 3.48 mm (Scotchcast masks) and 3.02 mm (thermoplastic) in 3 DOF. Differences between Scotchcast and thermoplastic masks were statistically significant (p < 0.001) in corrections allowing 6 DOF, but not in standard systems allowing 3 DOF (Table 2). Patients immobilized in Scotchcast masks however, did not differ in their baseline characteristics (i.e. with respect to age at radiotherapy) from patients immobilized in thermoplastic masks. There was a significant difference though in the number of position controls between 6 DOF and 3 DOF (p < 0.001).

Prior to on-board imaging becoming commonly available, our in-house protocols required an action level of 3 mm displacement in any direction (lateral, longitudinal, vertical) for offline-correction of weekly position checks in fractionated radiotherapy. Hence analysis of acquired position controls as to number of necessary position corrections with respect to the formerly established action level showed no differences in 3 DOF but significantly higher numbers for the thermoplastic systems in 6 DOF (3.0% vs. 23%, p < 0.001) (Table 3). However, there was also a significant difference between the number of required interventions if defined as deviation of > 3 mm in any direction or defined as deviation of the overall displacement vector in 3 DOF (3 DOF: p < 0.001; 6 DOF: p = 0.14). Considering the overall displacement vector in 3 DOF, misalignments could be significantly higher leading to a higher number of required interventions than consideration of the maximum translational deviation alone.

**Table 3:** Action levels

	6DOF		p =	3DOF		p =
Action Levels	Scotchcast	Thermoplast		Scotchcast	Thermoplast	
> 3 mm (component)	3%	23%	< 0.001	22%	15%	0.057
> 3 mm (radial vector)	7%	28%	< 0.001	53%	49%	0.448

## Discussion

Isocentre shifts of approximately 1 to 4 mm in this patient cohort representing set-up accuracy are within the expected range of extracranial targets in the head and neck [4,9,11-16,18,19,24]. Higher precision for the Scotchcast or thermoplastic systems have been reported before [15,17,24], however it needs to be mentioned that mostly intracranial targets were evaluated in systems allowing position correction in 3DOF. Treatment of extracranial targets with these immobilization systems has been investigated resulting in less accurate positioning of more distal as compared to intracranial targets [18,19]. This is supported by the clinicians' experience in everyday clinical routine in the conventional radiotherapy.

Reproducibility of fixation devices can be analyzed by evaluation of standard deviations of respective set-up corrections. In our cohort, this was evaluated by the root mean square of all patients' standard deviations or the centered distributions as described above.

In view of the higher rigidity of Scotchcast masks as opposed to thermoplastic head masks, higher reproducibility of the Scotchcast immobilization would initially be expected. This is supported by our data for 3 and 6 DOF except for the vertical component. The Scotchcast mask's rigid shell does not seem to allow significant motion in both the vertical and lateral direction but does allow some motion in the longitudinal direction. Thermoplastic head masks on the other hand immobilize the patient between headrest and thermoplastic layer with very little motion in the vertical direction. Less restriction apparently occurs in the lateral and longitudinal direction.

Scotchcast and thermoplastic (including shoulder fixation) masks were shown to immobilize head and neck cancer patients equally well if considering 3 DOF position correction only. Higher discrepancies were found when comparing these systems in 6 DOF. While these statistically significant differences could not be attributed to the patients' age distribution in the two immobilization groups, overall differences (Scotchcast and thermoplastic immobilization) were higher in the 3 DOF position correction versus 6 DOF which is supported by Spadea et al [25].

This difference was maintained presuming our traditional action level of 3 mm in fractionated head and neck treatments. Albeit isocentre localization was similar in 3 DOF and 6 DOF, target volumes usually extended more caudally in the 3 DOF (IMRT) as compared to 6 DOF (C12). Therapists had to consider adequate target position over a higher volume therefore making the best possible compromise for positioning while only the more cranial part (CTV1) of the CTV2 had to be considered in carbon ion therapy.

We are aware different imaging modalities were used for position verification in 3 DOF (MV-CBCT) as opposed to 6 DOF (orthogonal x-rays). However, various investigations have already been carried out addressing the issue of imaging modality for position verification suggesting orthogonal x-rays to be equivalent to CBCT for the determination of setup errors [2,15].

Also, we have analyzed significantly higher numbers of position checks in 3 DOF than in 6 DOF. This however, is due to the nature of our treatment regimen applying mostly 8 fractions of carbon ion therapy followed by approximately 25 fractions of IMRT for reported indications in head and neck malignancies.

The Scotchcast mask was shown to require lower absolute interfractional set-up corrections; hence, this fixation system appears superior for lesions in the vicinity of small critical structures such as optic nerves or the optic chiasm where the highest possible reproducibility is required.

In a rigid body setup such as our head and neck patients, optimal translational corrections were found to be dependent on whether or not rotations were included in the registration and position correction [26]. In standard treatments, where treatment tables commonly only allow corrections in 3 DOF without rotation correction capability, optimal corrections for translational shifts are dependent on registration landmarks. Therefore, it is recommended in rigid registrations to choose landmarks approximately coincident with the treatment site. Hence, when our therapists need to match the more extensive target volumes for IMRT following carbon ion treatment, compromises need to be made at the cranial/caudal edge of the target. Our findings practically illustrate these theoretical considerations of Murphy [26].

## Conclusion

Both fixation devices guarantee high reproducibility for patients with head and neck malignancies. Thermoplastic head masks including shoulder fixation also provide very good repositioning accuracy with additional immobilization the lower neck and presumably higher patient comfort. Scotchcast masks require lower interfractional set-up corrections though; therefore these are preferred if the highest possible reproducibility needs to be achieved.

While we have seen small expected repositioning errors in both of our mask systems, 6 DOF position verification reveals smaller positioning errors than 3 DOF. Radiation treatments requiring high positioning accuracy, image guidance still seems to be mandatory at each fraction in both systems to achieve best possible plan delivery and maintain optimal normal tissue sparing in particle therapy. If considering to define action levels for position correction, the overall displacement vector seems to be a more appropriate measure than the maximum translational error.

This, to our knowledge, is the first report directly comparing 6 DOF and 3 DOF position correction in a cohort of head and neck cancer patients for two commonly used immobilization systems.

## Competing interests

The authors declare that they have no competing interests.

## Authors' contributions

ADJ and MW as well as ON and MWM contributed equally. ADJ, JD, and MWM were responsible for treatment concepts, ADJ and MWM for target volume definition and treatment of the patients included, SPK for immobilization and SPK and MWM for immobilization concepts. MW and ON provided medical physics support (carbon ion treatment planning, quality assurance), ADJ, MW, ON, MWM collected and analysed the data presented here. All authors read and approved the final manuscript.
